# Course of clinical high-risk of psychosis in patients of an adult early detection service

**DOI:** 10.1192/j.eurpsy.2026.10375

**Published:** 2026-07-31

**Authors:** F. Schultze-Lutter

**Affiliations:** Department of Psychiatry and Psychotherapy, Heinrich-Heine University, Düsseldorf, Germany

## Abstract

**Introduction:**

The main focus of research on clinical high-risk states for psychosis (CHR) has been the development of criteria to predict psychosis. Consequently, patients not meeting CHR criteria but being referred to an early detection service as well outcomes other than psychosis have received less attention.

**Objectives:**

To investigate conversion rates as well as incidence, persistence, and remission rates of CHR states and their association with the different CHR approaches, i.e. symptomatic ultra-high risk and cognitive basic symptom criteria.

**Methods:**

Naturalistic follow-up (median 53.7 months, range 13.9– 123.7 months) of 246 outpatients of an early detection of psychoses service (21.1% CHR negative and 78.9% CHR positive).

**Results:**

Eighty-one patients (32.9%) developed a first-episode psychosis, mainly schizophrenia (75.3%). The remission rate of CHR status was 43.3% of all 194 CHR-positive cases, including converters, or 72.4% if only the 116 non-converters were considered. The persistence rate of any CHR criteria was 27.6%, and new occurrence rate in initially CHR-negative patients was 9.1%. At follow-up, 54.5% of the non-converters met criteria of at least one Axis-I diagnosis, mainly affective and anxiety disorders, and 68.4% had at least mild functional problems, whereby the presence of CHR criteria at follow-up (either newly developed or maintained) but not that of baseline CHR criteria was associated with significantly lower functioning.

**Conclusion:**

Over the average four-year follow-up, conversion-to-psychosis and remission-from-CHR-state rates were similar around one third. The presence of CHR criteria seems to maintain the risk for lower functioning and mental disorders, particularly for affective disorders, in non-converters. Thus, therapeutic efforts targeting CHR patients should also focus on comorbid mental disorders as well as social and role functions to improve the long-term outcome.

**Disclosure of Interest:**

None Declared

